# Egr-1 Activation by Cancer-Derived Extracellular Vesicles Promotes Endothelial Cell Migration via ERK1/2 and JNK Signaling Pathways

**DOI:** 10.1371/journal.pone.0115170

**Published:** 2014-12-12

**Authors:** Yae Jin Yoon, Dae-Kyum Kim, Chang Min Yoon, Jaesung Park, Yoon-Keun Kim, Tae-Young Roh, Yong Song Gho

**Affiliations:** 1 Division of Integrative Biosciences and Biotechnology, Pohang University of Science and Technology, Pohang 790-784, Republic of Korea; 2 Department of Life Sciences, Pohang University of Science and Technology, Pohang 790-784, Republic of Korea; 3 Department of Mechanical Engineering, Pohang University of Science and Technology, Pohang 790-784, Republic of Korea; 4 Ewha Institute of Convergence Medicine, Ewha Womans University Medical Center, Seoul 158-710, Republic of Korea; University of Nebraska Medical Center, United States of America

## Abstract

Various mammalian cells, including cancer cells, shed extracellular vesicles (EVs), also known as exosomes and microvesicles, into surrounding tissues. These EVs play roles in tumor growth and metastasis by promoting angiogenesis. However, the detailed mechanism of how cancer-derived EVs elicit endothelial cell activation remains unknown. Here, we provide evidence that early growth response-1 (Egr-1) activation in endothelial cells is involved in the angiogenic activity of colorectal cancer cell-derived EVs. Both RNA interference–mediated downregulation of Egr-1 and ERK1/2 or JNK inhibitor significantly blocked EV-mediated Egr-1 activation and endothelial cell migration. Furthermore, lipid raft-mediated endocytosis inhibitor effectively blocked endothelial Egr-1 activation and migration induced by cancer-derived EVs. Our results suggest that Egr-1 activation in endothelial cells may be a key mechanism involved in the angiogenic activity of cancer-derived EVs. These findings will improve our understanding regarding the proangiogenic activities of EVs in diverse pathological conditions including cancer, cardiovascular diseases, and neurodegenerative diseases.

## Introduction

Various types of mammalian cells, such as cancer cells, macrophages, endothelial cells, platelets, and epithelial cells release extracellular vesicles (EVs) into their surroundings from the plasma and endosomal membrane compartments [1]–[4]. These mammalian EVs, also known as exosomes and microvesicles, are spherical bilayered proteolipids with an average diameter of 40–250 nm and are enriched with various bioactive constituents, including proteins, lipids, and genetic material [1]–[9]. Growing evidence has revealed that EVs play pleiotropic functions in intercellular communication: EVs stimulate recipient cells by the activation of a receptor and the transfer of membrane proteins, signaling molecules, mRNAs, and miRNAs [4]–[9].

EVs have often been referred to as “cellular dust”, although cells shed EVs either constitutively or in a regulated manner [1]–[9]. Moreover, the proteins, mRNAs, or miRNAs in EVs differ in composition depending on the states of donor cells [1], [4]. Recently, our group revealed that proteins of human colorectal cancer cell-derived EVs are interconnected via physical interactions and cluster into functional modules involved in EV biogenesis and function [4], [10]. Furthermore, the secretion of EVs is a universal cellular process occurring from simple organisms (Archea or Gram-negative and Gram-positive bacteria) to complex multicellular organisms, suggesting that this EV-mediated communication is evolutionarily conserved [9], [11]–[13]. Taken together, these findings suggest that EVs play diverse roles in intercellular communication [6], [10]. However, the pathophysiological roles of EVs are not completely understood.

Angiogenesis, the formation of new blood vessels from preexisting vasculature, is a complex and multistep process involving adhesion, migration, invasion, proliferation, and differentiation of endothelial cells [14], [15]. This neovascularization occurs under various normal and pathological conditions [14]. For example, angiogenesis is essential for tumor growth and metastasis by providing oxygen and nutrients to the growing tumor [15]. In the tumor microenvironment, a heterogeneous population of cells, including cancer cells, endothelial cells, fibroblasts, and immune cells modulates an environment favorable to tumor growth and invasion [16]–[18]. These cancer and stromal cells secrete vascular endothelial growth factor (VEGF), fibroblast growth factor 2 (FGF2), tumor necrosis factor-α (TNF-α), and IL-6 into the surrounding area and these factors contribute to tumor-associated angiogenesis [16]–[19].

In addition to these proangiogenic soluble factors, the cells comprising the tumor tissue secrete EVs into the extracellular milieu and these shed EVs play multiple roles in tumor growth and metastasis by promoting angiogenesis, tumor invasion, and immune escape [4]–[8], [20]–[23]. After the initial report on the angiogenic activities of EVs derived from HT1080 human fibrosarcoma and DU-145 human prostate carcinoma cells [5], several studies confirmed that EVs derived from cancer cells, fibroblasts, and cancer stem cells promote *in vitro* and *in vivo* angiogenesis [4], [8], [24]–[28]. These angiogenic activities of EVs are mediated by vesicular lipid(s), proteins, including receptors and tetraspanin proteins, mRNAs, and miRNAs. However, the detailed mechanism of how EVs elicit angiogenic activity has not been extensively studied.

Early growth response-1 (Egr-1), an immediate early gene and a zinc finger transcription factor, plays a crucial role in angiogenesis [29]–[32]. In addition to serum exposure, Egr-1 can be rapidly and transiently induced by cytokine, growth factor, and environmental stress, including hypoxia, fluid shear stress, and vascular injury [33], [34]. Egr-1 regulates the expression of proangiogenic genes, such as VEGF, FGF2, and IL-6 in endothelial cells or TNF-α in macrophages [31], [34]–[36]. Within the tumor tissue, endothelial cells, cancer cells, fibroblasts, and tumor-infiltrating macrophages can express Egr-1. Furthermore, microvessel densities in tumor tissues obtained from Egr-1-deficient mice are lower than those obtained from wild-type mice [37] and vessel-like structure formation in tumor tissue was suppressed by DNAzymes that target Egr-1 mRNA [31], suggesting that Egr-1 plays essential roles in tumor growth and angiogenesis. In this regard, several studies have reported that Egr-1 expression in cancer cells, endothelial cells, and macrophages is related to tumor progression [32], [36]–[38]. Collectively, these findings suggest that Egr-1 plays important roles in tumor-associated angiogenesis and tumor progression.

In this report, we provide evidence that Egr-1 activation in endothelial cells should be a key mechanism involved in the angiogenic activity of cancer-derived EVs. We found that Egr-1 activation by colorectal cancer cell-derived EVs promoted endothelial cell migration via the ERK1/2 and JNK signaling pathways and lipid raft-mediated endocytosis.

## Materials and Methods

### Cell culture

Human colorectal adenocarcinoma (SW480), colorectal carcinoma (HCT116), lung adenocarcinoma (A549), and fibrosarcoma (HT1080), and normal bronchial epithelial (BEAS-2B) cells were maintained in RPMI 1640 (Invitrogen, Carlsbad, CA, USA) supplemented with 10% fetal bovine serum (FBS; Invitrogen), 100 U/mL penicillin, and 0.1 mg/mL streptomycin. Human neuroblastoma (SH-SY5Y) and prostate carcinoma (PC3) cells were maintained in MEM (Invitrogen) supplemented with 10% FBS, 100 U/mL penicillin, and 0.1 mg/mL streptomycin. SW480, HCT116, A549, HT1080, SH-SY5Y, PC3, and BEAS-2B were purchased from American Type Culture Collection. Human microvascular endothelial cells (HMEC-1s) were cultured in Endothelial Growth Medium-2 (EGM-2; Lonza, Walkersville, MD, USA) [5]. Human umbilical vein endothelial cells (HUVECs) were isolated from freshly delivered umbilical cords and maintained as described previously [39]. HUVECs were cultured in medium 199 (Invitrogen) supplemented with 20% FBS, 3 ng/mL FGF2 (R&D Systems, Minneapolis, MN, USA), 5 U/mL heparin (Sigma-Aldrich, St Louis, MO, USA), 100 U/mL penicillin, and 0.1 mg/mL streptomycin. Cells were cultured at 37°C in a humidified atmosphere of 5% CO_2_. All cell lines were mycoplasma-free confirmed using e-MyCo Mycoplasma PCR Detection Kit (iNtRON Biotechnology. Inc., Seoul, Korea).

### Purification of EVs

EVs were purified by a combination of differential centrifugation, ultrafiltration using a 100-kDa hollow fiber membrane, ultracentrifugation onto sucrose cushions, and iodixanol density gradient ultracentrifugation according to previously established methods [8]. Briefly, 80–90% confluent cells were washed twice with phosphate buffered saline and then were incubated for 24 h in serum-free RPMI 1640 medium (SW480, HCT116, A549, HT1080, and BEAS-2B) or serum-free MEM medium (SH-SY5Y and PC3). Approximately 2,000 mL of conditioned medium was centrifuged at 500 *g* for 10 min, and then twice at 2,000 *g* for 15 min to eliminate cell contamination. Supernatant was further concentrated using the QuixStand Benchtop System with a 100-kD hollow fiber membrane (GE Healthcare, Piscataway, NJ, USA). The concentrate (∼35 mL) was placed upon 0.5 mL of 0.8 and 2.0 M sucrose cushions in buffer (20 mM HEPES, 150 mM NaCl, pH 7.4) and then centrifuged at 100,000 *g* for 2 h (SW 32 Ti swing bucket rotor with a k-factor of 256.8). The EVs (0.5 mL) were harvested from the interface between the 0.8 and 2.0 M sucrose cushions, diluted with 9 mL of phosphate buffered saline, placed upon 0.35 mL of 0.8 M and 0.15 mL of 2.0 M sucrose cushions, and centrifuged at 100,000 *g* for 2 h (SW 41 Ti swing bucket rotor with a k-factor of 256.6). The EVs (0.5 mL) were harvested from the interface between the 0.8 and 2.0 M sucrose cushions, and were diluted with 1.42 mL of phosphate buffered saline and 2.88 mL of 50% iodixanol (Axis-Shield PoC AS, Nycomed, Norway), to give 30% iodixanol. This sample was placed at the bottom of a tube and overlaid with 3 mL of 20% iodixanol and 2.5 mL of 5% iodixanol. After centrifugation at 200,000 *g* for 2 h (SW 41 Ti swing bucket rotor with a k-factor of 128.3), 10 fractions of equal volume (1 mL) were removed from the top of the gradient. Finally, the purified EVs were measured for their protein content using the Bradford protein assay (Bio-Rad, Munich, Germany) and applied to further assays. We obtained 70–150 µg of EVs in total proteins from ∼2,000 mL of 24 h serum-free conditioned medium of cancer cells (SW480, HCT116, A549, HT1080, SH-SY5Y, and PC3): the yield of EVs from the same amount of normal human bronchial epithelial cells (BEAS-2B) is ∼10 µg in total proteins.

### Western blot analysis

Each fraction of iodixanol density gradients was separated by SDS-PAGE and then transferred to a polyvinylidene difluoride membrane. The membrane was blocked and incubated with mouse anti-CD81 (BD Biosciences, San Jose, CA), mouse anti-CD63 antibody (Santa Cruz Biotechnology, Santa Cruz, CA), mouse anti-GM130 antibody (BD Biosciences), and mouse anti-cytochrome *c* antibody (BD Biosciences), followed by goat anti-mouse antibodies conjugated to horseradish peroxidase (Santa Cruz Biotechnology). The immunoreactive bands were visualized with a chemiluminnescent substrate.

### Murine Matrigel assay and immunostaining

We used 6-8 week old wild-type mice from the Jackson Laboratory. Mice (n = 5) were subcutaneously injected with 0.5 mL Matrigel (BD Biosciences) containing 20 µg of SW480-derived EVs or phosphate buffered saline (Invitrogen). On day 7 after injection, the mice were killed, and the Matrigel was removed and stained for whole-mount immunofluorescence. Blood vessels were immunostained with goat anti-CD31 antibody (Cell Signaling Technology) followed by AlexaFluor488 donkey anti-goat antibody (Invitrogen). All images were visualized using an FV1000 Olympus confocal microscope (Olympus, Tokyo, Japan) equipped with a UPlanSApo 20×/0.75 objective lens and acquired using FV1000-ASW 1.5 software (Olympus). The CD31-positive area (µm^2^) in the immunofluorescence image was quantitatively analyzed using ImageJ software (National Institutes of Health). All animals received humane care, and the experiments were approved by the Institutional Animal Care and Use Committee at Pohang University of Science and Technology, Pohang, Republic of Korea (approval number: 2011-01-0015).

### Scratch wound-healing assay

HMEC-1s were plated in 24-well cell-culture plates (Corning Inc., Corning, NY) at a density of 1×10^5^ cells per well and allowed to attach overnight. Confluent HMEC-1s were injured by a deliberate scratch and incubated with control, SW480-derived EVs (1 µg/mL, 0.5 mL), or EGM-2. Twelve hours after injury, cells were washed in phosphate buffered saline, stained with CellTracker (Invitrogen), and fixed in 4% paraformaldehyde before fluorescence imaging. Images were visualized under an Olympus 1×81 inverted fluorescence microscope (Olympus) and acquired using MetaMorph software (Molecular Devices, Sunnyvale, CA, USA).

### Endothelial proliferation assay

HMEC-1s were plated in 24-well cell-culture plates (Corning Inc., Corning, NY) with glass coverslips (Fisher Scientific, Rochester, NY) at a density of 5×10^4^ cells per well and allowed to attach overnight. After a 24 h treatment with control, SW480-derived EVs (1 µg/mL, 0.5 mL), or EGM-2, cells were fixed with 4% paraformaldehyde and incubated with rabbit anti-phospho-histone H3 (PH3) antibody (Upstate Biotechnology, Lake Placid, NY) followed by AlexaFluor488 goat anti-rabbit IgG antibody (Invitrogen). Nuclei were counterstained with Hoechst (Sigma-Aldrich). Images were visualized under an FV1000 Olympus confocal microscope (Olympus) and acquired using FV1000-ASW 3.0 software (Olympus). The percentage of PH3-positive cells was quantified by counting the cells with co-localized fluorescence signals.

### Real-time RT-PCR

PCR primers used in this study were designed using the Primer3 program (Whitehead Institute, http://biotools.umassmed.edu/bioapps/primer3_www.cgi). The primers used for gene amplification were as follows: GAPDH forward, 5′-CGAGATCCCTCCAAAATCAA-3′; GAPDH reverse, 5′-TTCACACCCATGACGAACAT-3′; EGR1 forward, 5′-CCGCAGAGTCTTTTCCTGAC-3′; EGR1 reverse, 5′-AGCGGCCAGTATAGGTGATG-3′. HMEC-1s (2×10^5^ cells) plated in 6-well cell-culture plate were treated with EVs (1 µg/mL, 2.0 mL) for 0.5, 1, 1.5, 2 and 4 h. RNA was extracted from cultured cells using the RNeasy Mini Kit (QIAGEN, Valencia, CA, USA). For real time RT-PCR, total RNA (100 ng) was amplified with a One Step SYBR RT-PCR Kit (TaKaRa Bio, Tokyo, Japan) using a LightCycler 2.0 PCR system (Roche Diagnostics, Mannheim, Germany). Amplification was carried out by heating the samples to 50°C for 2 min, then at 95°C for 10 min, followed by repeating cycles at 95°C for 15 sec, 55°C for 10 sec, and 72°C for 10 sec, for a total 45 cycles. The comparative Ct method was used for relative quantification of target gene expression against that of a housekeeping gene, GAPDH [40].

### Egr-1 nuclear translocation

HMEC-1s were plated in 24-well cell-culture plates (Corning Inc.) with glass coverslips (Fisher Scientific) at a density of 5×10^4^ cells per well and allowed to attach overnight. Cells were treated with SW480-derived EVs (1 µg/mL, 0.5 mL), fixed with 4% paraformaldehyde, and incubated with rabbit anti-Egr-1 antibody (Cell Signaling Technology, Hitchin, United Kingdom) followed by AlexaFluor488 goat anti-rabbit IgG antibody (Invitrogen). Nuclei were counterstained with Hoechst (Sigma-Aldrich). Images were visualized under an FV1000 Olympus confocal microscope (Olympus) and acquired using FV1000-ASW 3.0 software (Olympus). The percentage of Egr-1-positive cells was quantified by counting the cells with co-localized fluorescence signals.

To investigate the effect of signaling inhibitors (BioMol Research Laboratories, Plymouth Meeting, PA, USA) or methyl-β-cyclodextrin (MβCD; Sigma-Aldrich) on Egr-1 nuclear translocation, HMEC-1s were treated with ERK1/2 inhibitor (PD98059, 20 µM), p38 MAPK inhibitor (SB203580, 10 µM), JNK inhibitor (SP600125, 20 µM), Akt inhibitor (BML-257, 20 µM), or MβCD (10 mM) in the presence or absence of SW80-derived EVs (1 µg/mL).

### RNA interference-mediated downregulation of Egr-1

Small interfering RNA (siRNA) of Egr-1 (Bioneer, Daejeon, Korea) to a final concentration of 50 nM was transfected into HMEC-1s using Welfect-Q (Welgene, Taegu, Korea). Non-silencing scrambled siRNA was used as the negative control. After 48 h, cells were used for real-time RT-PCR analysis to determine the level of Egr-1 mRNA expression, Egr-1 nuclear translocation assays, and scratch wound-healing assays. The siRNA sequences were as follows: scrambled siRNA, 5′-CCUACGCCACCAAUUUCGU-3′; Egr-1 siRNA-1, 5′-CAGUAUCAUCUCCAUCAUA-3′; Egr-1 siRNA-2, 5′-AGUUUGCCAGGAGCGAUGA-3′; Egr-1 siRNA-3, 5′-GUGCAAUUGUGAGGGACAU-3′.

### EV uptake

SW480-derived EVs were labeled with DiI (Invitrogen) according to the manufacturer's instructions. Briefly, SW480-derived EVs (100 µg) were incubated with DiI (1 µM) for 15 min at 37°C and centrifuged at 100,000 *g* for 2 h at 4°C (Type 90 Ti fixed angle rotor with a k-factor of 126.4). The pellets, DiI-labeled EVs, were washed with phosphate buffered saline, and after ultracentrifugation at 100,000 *g* for 2 h at 4°C (Type 90 Ti fixed angle rotor with a k-factor of 126.4), were resuspended in phosphate buffered saline. HMEC-1s were pretreated without or MβCD (10 mM) for 0.5 h, and then incubated with DiI-labeled SW480-derived EVs (1 µg/mL, 0.5 mL) for 1 h. Cells were then fixed with 4% paraformaldehyde and nuclei were stained with Hoechst (Sigma-Aldrich). Images were visualized under an FV1000 Olympus confocal microscope (Olympus) and acquired using FV1000-ASW 3.0 software (Olympus).

### Statistical analyses

All values are expressed as means ± S.D. *P* values were calculated from one-way or two-way analysis of variance (ANOVA) with Bonferroni correction, based on comparisons with the appropriate control samples tested at the same time.

## Results

### SW480-derived EVs promote *in vivo* and *in vitro* angiogenesis

**Figure 1:**
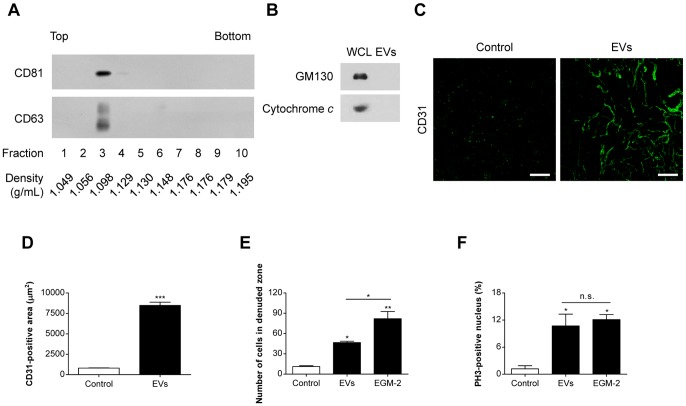
*In vivo* and *in vitro* angiogenesis induced by SW480-derived EVs. (A, B) EVs released by SW480 cells were purified from culture supernatants by a combination of differential centrifugation, ultracentrifugation onto sucrose cushions, and iodixanol density gradients. Each fraction of iodixanol density gradients was analyzed by Western blotting to detect CD81 and CD63, marker proteins of EVs (A). The purified EVs in fraction 3 were analyzed by Western blotting to detect non-EV marker proteins (GM130 and cytochrome *c*). SW480-derived whole cell lysate (WCL; 10 µg) and SW480-derived EVs (EVs; 10 µg) were loaded for Western blotting analysis (B). (C, D) Matrigel in the presence or absence of SW480-derived EVs (20 µg) was injected subcutaneously into C57BL/6 mice. After 7 days, whole-mount staining of Matrigel with anti-CD31 antibody was conducted (n = 5). Representative confocal Z-stack photographs of whole mounts stained for CD31 (green) are shown in panel C. Scale bars represent 100 µm. Fluorescence intensities of CD31 staining of the Z-stack plane of the Matrigel were measured as described in the Methods (D). (E) Migratory activity of SW480-derived EVs was evaluated by a scratch wound-healing assay. Confluent HMEC-1s were scratched and treated with SW480-derived EVs (1 µg/mL); then the number of migrated cells in the denuded zone was evaluated after 12 h (n = 3). (F) Proliferative activity of SW480-derived EVs (1 µg/mL) was evaluated by assessing the mitosis marker PH3. After 24 h, the PH3 and nuclei were stained with anti-PH3 antibody and Hoechst, respectively and evaluated by confocal microscopy. The percentage of PH3-positive cells in HMEC-1s was quantified by counting the cells with co-localized fluorescence signals (n = 3). As a positive control, HMEC-1s were treated with EGM-2 medium supplemented with diverse angiogenic factors such as EGF, FGF2, VEGF, and IGF1. Data are represented as mean ± SD. *, *P*<0.05; **, *P*<0.01; ***, *P*<0.001; n.s., not significant.

EVs released by SW480 cells were purified from culture supernatants by a combination of differential centrifugation, ultrafiltration using a 100-kDa hollow fiber membrane, ultracentrifugation onto sucrose cushions, and iodixanol density gradients as reported [8]. Consistent with the previous study [8], the purified EVs had a density of ∼1.098 g/mL and harbored CD81 and CD63, EV marker proteins (Fig. 1A). However, these purified EVs did not contain GM130 (a *cis*-Golgi protein) and cytochrome *c* (a mitochondrial protein found in apoptotic bodies): these two proteins are well-known non-vesicular proteins (Fig. 1B). We first showed that EVs derived from SW480 human colorectal adenocarcinoma cells induced *in vivo* neovascularization (Fig. 1C and 1D). When SW480-derived EVs within Matrigel were injected subcutaneously into mice, a massive formation of CD31-positive vessel-like structures was observed (Fig. 1C). In contrast, no apparent vessel formation was detected in the Matrigel without EVs. EVs significantly induced a 10.4-fold increase in the CD31-positive area in Matrigel when compared with the untreated control (Fig. 1D).

We next investigated the proangiogenic activity of SW480-derived EVs on human endothelial cells, HMEC-1s *in vitro*. Scratch wound-healing assays revealed that EVs induced a 4.1-fold increase in the number of migrated endothelial cells into the denuded zone compared with the untreated control (Fig. 1E). Furthermore, EVs potently stimulated the proliferation of endothelial cells: the percentage of PH3-positive cells increased 8.9-fold over that of the unstimulated cells (Fig. 1F). The positive control, EGM-2 supplemented with diverse angiogenic factors such as EGF, FGF2, VEGF, and IGF1, also induced both endothelial cell migration and proliferation. The migratory and proliferative activities of SW480-derived EVs were also observed in other endothelial cells, HUVECs (data not shown). Therefore, our data indicate that SW480-derived EVs have *in vivo* and *in vitro* angiogenic activities.

### SW480-derived EVs induce Egr-1 activation in endothelial cells

**Figure 2:**
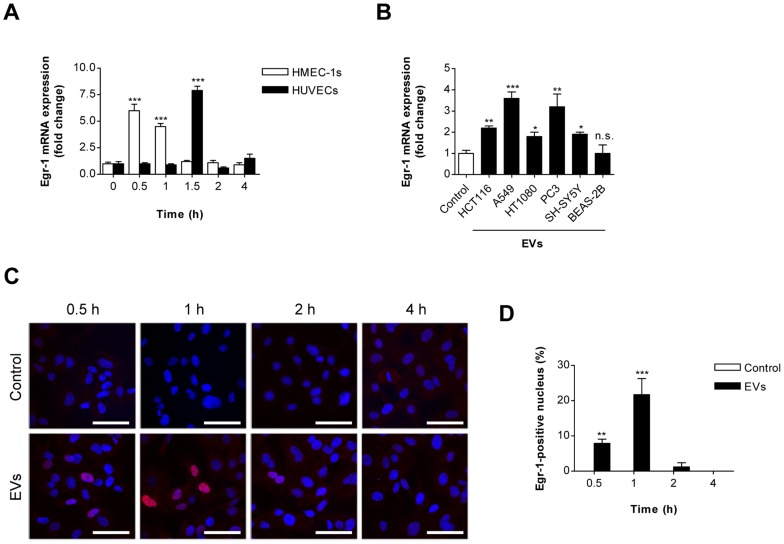
Egr-1 activation in endothelial cells by SW480-derived EVs. (A) HMEC-1s and HUVECs were incubated with SW480-derived EVs (1 µg/mL) or untreated control. mRNA was isolated from untreated control cells or cells treated with EVs for 0, 0.5, 1, 2, and 4 h and analyzed using real time RT-PCR (n = 3). Values represent Egr-1 mRNA/GAPDH mRNA normalized to untreated control cells. (B) HMEC-1s were treated with EVs (1 µg/mL) derived from HCT116 colorectal carcinoma, A549 lung adenocarcinoma, HT1080 fibrosarcoma, PC3 prostate carcinoma, SH-SY5Y neuroblastoma, and BEAS-2B normal bronchial epithelial cells for 0.5 h (n = 3). (C, D) In HMEC-1s, nuclear translocation of Egr-1 protein after stimulation with SW480-derived EVs (1 µg/mL) for 0.5, 1, 2, and 4 h was analyzed under confocal microscopy (n = 3). Nuclei and Egr-1 proteins were stained with Hoechst (blue) and anti-Egr-1 antibody (red), respectively. Co-localized fluorescence signals (purple) indicate the translocation of Egr-1 into the nucleus. Representative photographs are shown in panel C. Scale bars represent 30 µm. The percentage of Egr-1-positive nuclei was determined by measuring the number of cells with nucleus colocalized signals over that of total cells (D). Data are represented as mean ± SD. *P<0.05; **P<0.01; **P<0.001; n.s., not significant.

Egr-1 plays a crucial role in tumor-associated angiogenesis [29]–[32]. We thus examined the possibility of activation of endothelial Egr-1 by SW480-derived EVs (Fig. 2). Real time RT-PCR analyses revealed that treatment with SW480-derived EVs caused rapid and transient elevation of Egr-1 expression at the transcriptional level in human endothelial cells, HMEC-1s and HUVECs (Fig. 2A). We used HMEC-1s in the following experiments. Moreover, EVs from other human cancer cells (HCT116 colorectal carcinoma, A549 lung adenocarcinoma, HT1080 fibrosarcoma, PC3 prostate carcinoma, and SH-SY5Y neuroblastoma) also induced Egr-1 mRNA expression in human endothelial cells, whereas EVs from normal human bronchial epithelial cells (BEAS-2B) did not (Fig. 2B). We further investigated Egr-1 activation after stimulation by SW480-derived EVs. EVs induced rapid and transient expression and nuclear translocation of Egr-1 protein in HMEC-1s; the maximum effect was observed at 1 h after EV treatment (Fig. 2C and 2D). Taken together, cancer-derived EVs induce Egr-1 activation by increasing its expression and nuclear translocation in endothelial cells. These observations suggest that Egr-1 activation might be critical for modulating EV-induced angiogenesis.

### Egr-1 siRNA attenuates endothelial cell migration induced by SW480-derived EVs

**Figure 3:**
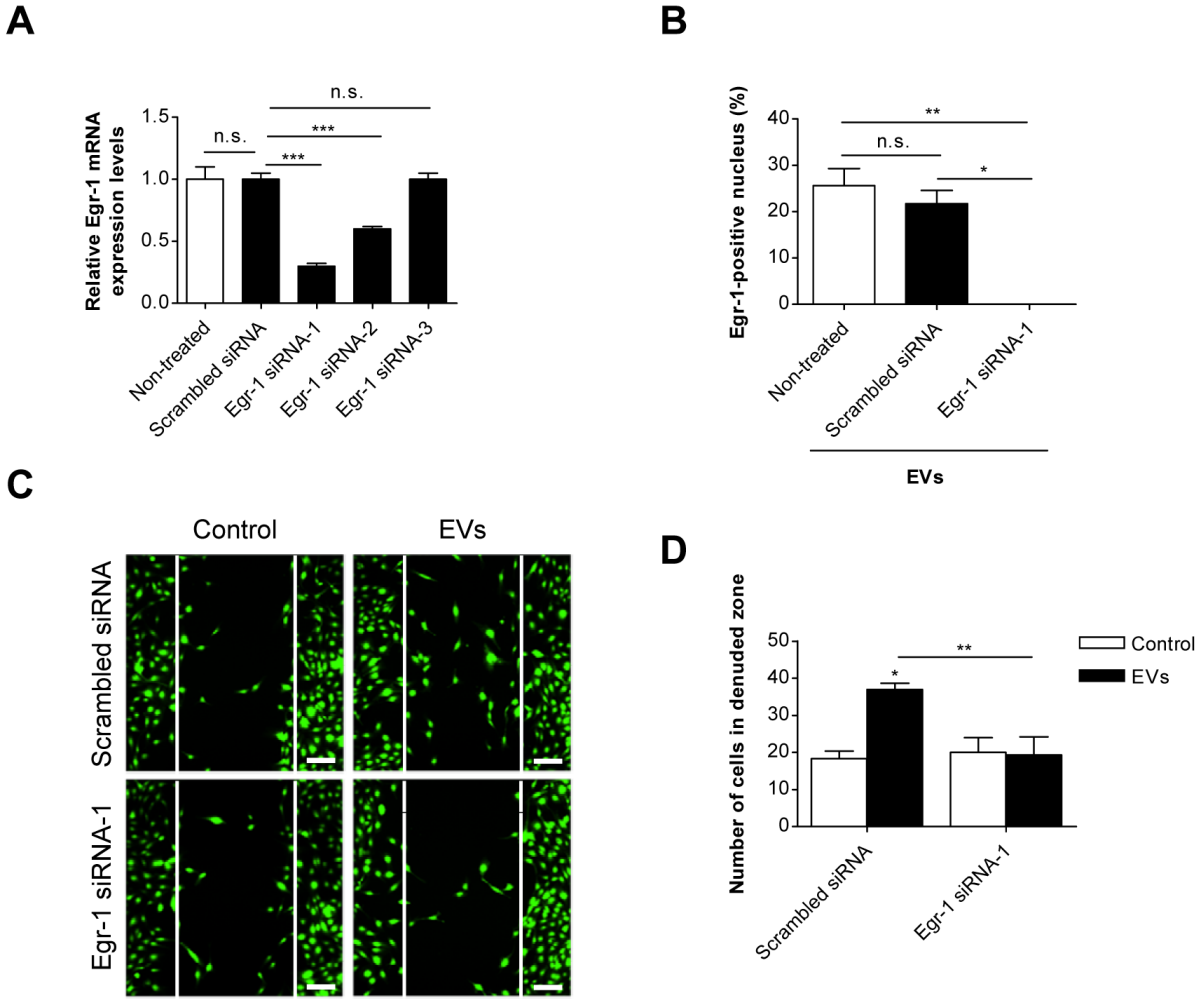
Inhibition of Egr-1 activation and endothelial cell migration by Egr-1 siRNA. (A) HMEC-1s were transfected with 50 nM of scrambled siRNA or Egr-1 siRNA-1, Egr-1 siRNA-2, or Egr-1 siRNA-3. mRNAs were isolated from the cells after 48 h and the level of Egr-1 mRNA was analyzed using real time RT-PCR (n = 3). (B) Nuclear translocation of Egr-1 protein after stimulation with SW480-derived EVs (1 µg/mL) for 1 h was analyzed in scrambled siRNA (50 nM) or Egr-1 siRNA-1 (50 nM) transfected HMEC-1s (n = 3). (C, D) Confluent HMEC-1s transfected with scrambled siRNA (50 nM) or Egr-1 siRNA-1 (50 nM) were scratched and treated with SW480-derived EVs (1 µg/mL); then the number of migrated cells in the denuded zone was evaluated after 12 h (n = 3). The number of migrated cells in the denuded zone of each group is shown in panel D. Scale bars represent 100 µm. Data are represented as mean ± SD. *P<0.05; **P<0.01; *** *P*<0.001; n.s., not significant.

We next investigated the role of Egr-1 in EV-induced endothelial cell migration using siRNA. When we examined three Egr-1 siRNAs, we found that Egr-1 siRNA-1 most effectively reduced the Egr-1 mRNA level in endothelial cells but scrambled siRNA did not shown this inhibitory effect (Fig. 3A). Endothelial cells treated with Egr-1 siRNA-1 efficiently blocked EV-induced Egr-1 activation (Fig. 3B) and migration as observed in scratch wound-healing assays (Fig. 3C and 3D) while scrambled siRNA did not show these inhibitory effects. Thus, our results indicated that EV-induced expression and nuclear translocation of Egr-1 in endothelial cells should contribute to EV-induced endothelial cell migration.

### ERK1/2 and JNK signaling pathways are involved in EV-induced Egr-1 activation and migration in endothelial cells

**Figure 4:**
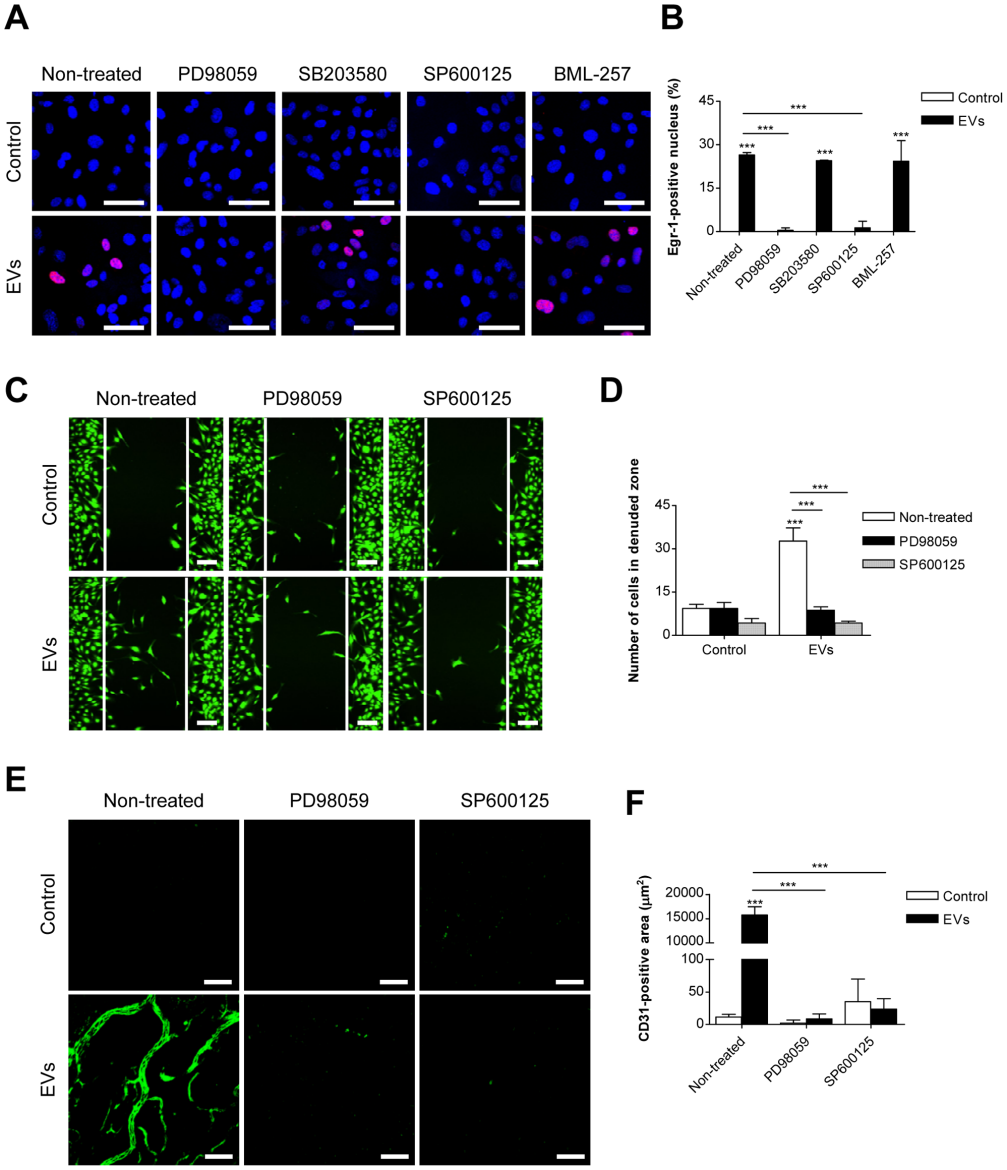
Role of ERK1/2 and JNK signaling pathways in SW480-derived EV-mediated endothelial cell migration. (A, B) HMEC-1s were pretreated with signaling inhibitors for 1 h and then stimulated for 1 h with SW480-derived EVs (1 µg/mL). Nuclear translocation of Egr-1 protein was analyzed using confocal microscopy (n = 3). Nuclei and Egr-1 proteins were stained with Hoechst (blue) and anti-Egr-1 antibody (red), respectively. Co-localized fluorescence signals (purple) indicate the translocation of Egr-1 into the nucleus. Representative photographs are shown in panel A. The percentage of Egr-1-positive nuclei was determined by measuring the number of cells with nucleus colocalized signals over total cells (B). (C, D) Confluent HMEC-1s were scratched and treated with SW480-derived EVs (1 µg/mL) in the presence or absence of signaling inhibitors; then the number of migrated cells in the denuded zone was evaluated after 12 h (n = 3). Representative photographs of confocal microscopic imaging are shown in panel C and the number of migrated cells in the denuded zone of each group is shown in panel D. ERK1/2 inhibitor, PD98059 (20 µM); p38 MAPK inhibitor, SB203580 (10 µM); JNK inhibitor, SP600125 (20 µM); Akt inhibitor, BML-257 (20 µM). (E, F) C57BL/6 mice were subcutaneously injected with Matrigel containing SW480-derived EVs (20 µg) with PD98059 (20 µM) or SP600125 (20 µM). After 7 days, whole-mount staining of Matrigel with anti-CD31 antibody was conducted (n = 5). Representative confocal Z-stack photographs of whole mounts stained for CD31 (green) are shown in panel E. Fluorescence intensities of CD31 in the Z-stack plane of the Matrigel were measured as described in the Methods (F). Scale bars in panels A, C, and E represent 30, 100, and 100 µm, respectively. Data are represented as mean ± SD. ***P<0.001.

We next investigated the signaling pathways involved in EV-mediated Egr-1 activation. ERK1/2 inhibitor (PD98059) and JNK inhibitor (SP600125) almost completely suppressed Egr-1 nuclear translocation in endothelial cells after stimulation with SW480-derived EVs, but p38 MAPK inhibitor (SB203580) and Akt inhibitor (BML-257) had no effect (Fig. 4A and 4B). Moreover, we observed that PD98059 and SP600125 treatments almost completely blocked EV-induced endothelial cell migration while these inhibitors showed no apparent effect on the basal migration of endothelial cells (Fig. 4C and 4D). Furthermore, PD98059 or SP600125 almost completely inhibited the *in vivo* angiogenic activities of SW480-derived EVs (Fig. 4E and 4F). All these observations indicated that the ERK1/2 and JNK signaling pathways are essential for Egr-1 activation, endothelial cell migration, and *in vivo* angiogenesis.

### Endocytosis inhibitor inhibits Egr-1 activation and endothelial cell migration induced by SW480-derived EVs

**Figure 5:**
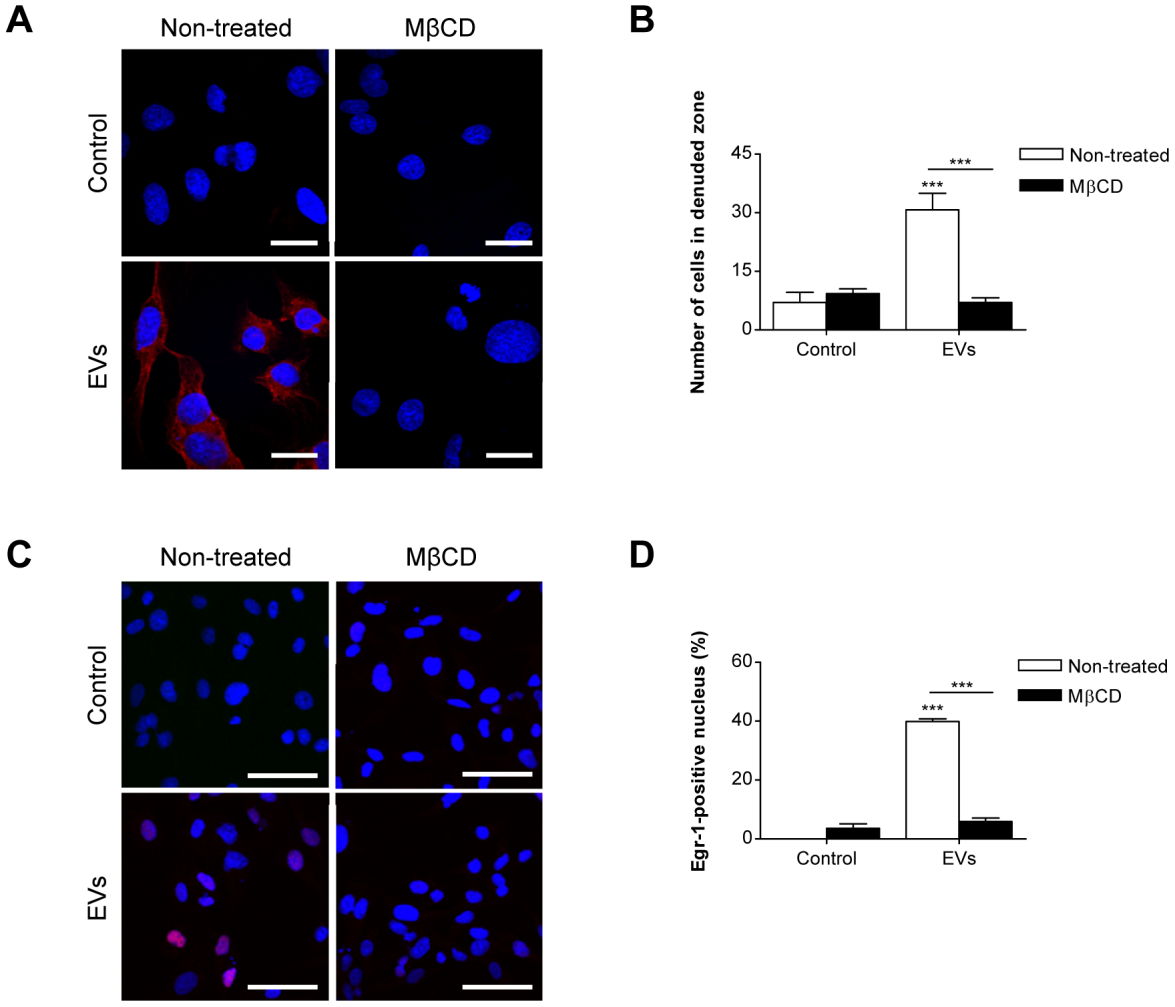
Inhibition of Egr-1 activation and endothelial cell migration by MβCD. (A) HMEC-1s were treated with DiI-labeled SW480-derived EVs (1 µg/mL) for 1 h in the presence or absence of MβCD (10 mM) (n = 3). SW480-derived EVs and nuclei were stained with DiI (red) and Hoechst (blue) respectively. Representative photographs are shown in panel A. Scale bars represent 10 µm. (B) Confluent HMEC-1s were scratched and treated with SW480-derived EVs (1 µg/mL) in the presence or absence of MβCD (10 mM); then the number of migrated cells in the denuded zone was evaluated after 12 h (n = 3). (C, D) HMEC-1s were pretreated with MβCD (10 mM) for 1 h and then stimulated with SW480-derived EVs (1 µg/mL) for 1 h. Scale bars represent 30 µm. Nuclear translocation of Egr-1 protein was analyzed using confocal microscopy and the percentage of Egr-1-positive nuclei was determined by measuring the number of cells with nucleus co-localized signals over that of total cells (n = 3). Data are represented as mean ± SD. ***P<0.001.

As a potential involvement of lipid raft endocytosis in EV uptake [41], [42], we examined the role of the lipid rafts in EV uptake by endothelial cells. DiI-labeled EV uptake was significantly inhibited following treatment with MβCD (10 mM) (Fig. 5A). EV-induced endothelial cell migration into denuded zone was completely blocked by MβCD treatment (Fig. 5B). Moreover, endothelial cells treated with MβCD completely blocked EV-induced Egr-1 activation (Fig. 5C and 5D). Thus, our results indicated that EV uptake via lipid rafts contributes to Egr-1 activation and migration in endothelial cells.

## Discussion

Although cancer-derived EVs play a pivotal role in tumor growth and metastasis by promoting angiogenesis [4]–[8], [24]–[27], the detailed mechanism of how cancer-derived EVs elicit endothelial cell activation is not completely understood. In the present study, we provide evidence that Egr-1 activation in endothelial cells is a key mechanism involved in the angiogenic activities of cancer-derived EVs. More specifically, we showed that: (1) SW480-derived EVs promoted *in vivo* and *in vitro* angiogenesis; (2) SW480-derived EVs induced Egr-1 activation by increasing its expression and nuclear translocation in endothelial cells; (3) EVs from other human cancer cells also induced Egr-1 expression in endothelial cells; (4) siRNA-mediated downregulation of Egr-1 and treatment with ERK1/2 or JNK inhibitor suppressed both EV-mediated Egr-1 activation and endothelial cell migration; and (5) Lipid raft-mediated endocytosis inhibitor, MβCD effectively inhibited endothelial Egr-1 activation and migration induced by SW480-derived EVs. Taken together, these data indicate that Egr-1 activation in endothelial cells is a crucial mechanism involved in cancer-derived EV-induced angiogenesis.

Egr-1 is a rapidly and transiently inducible gene by diverse stimuli, including growth factors, cytokines, and environmental stresses [29]–[32]. Transient Egr-1 expression patterns are observed in diverse Egr-1 producing cells such as endothelial cells, macrophages, and fibroblasts. For example, TNF-α and VEGF induced transient Egr-1 activation in HUVECs, with a maximum at 1 h and dropped to basal levels within 4 h [43]. However, sustained expression of Egr-1 suppresses tumor growth and neovascularization [32]. Sustained Egr-1 expression in endothelial cells led to strong upregulation of the corepressor NAB2 and other genes involved in anti-angiogenesis, growth arrest, and apoptosis [32]. Therefore, rapid and transient Egr-1 gene expression is crucial for tumor-associated angiogenesis and tumor progression.

In the present study, we observed that EVs derived from SW480 human colorectal cancer cells are Egr-1-inducible stimuli in endothelial cells, HMEC-1s and HUVECs. Egr-1 gene expression in these cells was transiently induced with maximal levels at 0.5–1.5 h and decreased to basal levels at 4 h after stimulation with SW480-derived EVs. Moreover, we showed that siRNA-mediated downregulation of Egr-1 significantly blocked EV-mediated endothelial cell migration by inhibiting EV-induced Egr-1 activation. Thus, rapid and transient regulation of Egr-1 expression and activation after stimulation with cancer-derived EVs should contribute to EV-induced endothelial cell activation and *in vitro* angiogenesis. However, further studies on Egr-1 knockout mice [44], [45] should be carried out to understand the exact role of endothelial Egr-1 during cancer EV-induced neovascularization. In addition, we found that endothelial Egr-1 expression can also be induced by EVs from other human cancer cells (HCT116 colorectal carcinoma, A549 lung adenocarcinoma, PC3 prostate carcinoma, HT1080 fibrosarcoma, and SH-SY5Y neuroblastoma) but not by normal human bronchial epithelial BEAS-2B cell-derived EVs. Although further study is required, we can speculate that Egr-1 expression in endothelial cells is a specific process regulated by cancer-derived EVs rather than a general phenomenon.

Recently, EVs were reported to harbor mRNAs that can be transferred to and function in the recipient cells by translation of vesicular mRNAs into proteins [7], [8]. Our previous study showed that SW480-derived EVs harbor 11,327 mRNAs: cell cycle-related mRNAs belonging to the M-phase are specifically enriched [8]. A further examination of our previous study, Egr-1 mRNA is also present in SW480-derived EVs. By quantitative real-time RT-PCR analysis, we found that the amount of Egr-1 mRNA present in 1 µg of EVs was equivalent to that of 5,800±500 HMEC-1s. Note that treatment with 2.0 µg of EVs to 2.0×10^5^ HMEC-1s in 2.0 mL culture medium caused the maximum 4.5- to 6.0-fold increase in Egr-1 mRNA levels compared with untreated controls: the amount of Egr-1 mRNA present in untreated control cells is about 17 times more than that of EVs. If we assume that all added EVs were internalized by cultured cells, a single HMEC-1 cell uptakes less than 10 pg of EVs in total protein amount and one seventeenth of Egr-1 mRNA molecules present in the untreated control cells. Thus, most of the Egr-1 mRNA increase in EV-treated endothelial cells should be due to the induction of Egr-1 expression at the transcriptional level rather than the transfer of vesicular Egr-1 transcripts to the cells. Although we do not know the local concentration of EVs in tumor tissues, the concentration of EVs in serum of cancer patients has been reported to be 1,000 µg/mL [46]. We can speculate that the local EV concentration in the tumor microenvironment would be higher than that in the serum, suggesting that EV dose used in this study (1 µg/mL) is patho-physiologically possible. Further study to determine the concentration of EVs in tumor tissues and to quantitate how many EVs are delivered to the target cell may help us understand the role of cancer EVs in tumor-associated angiogenesis.

In the present study, we demonstrated that siRNA-mediated downregulation of Egr-1 blocked EV-mediated Egr-1 activation and endothelial cell migration. Further dissection of the signaling pathways involving a pharmacologic approach revealed that ERK1/2 and JNK MAPKs, but not p38 MAPK or Akt pathways were crucial in the EV-mediated Egr-1 activation in endothelial cells. In agreement with our findings, the requirement of ERK1/2 and JNK activation for Egr-1 activation was also reported in cocaine-treated human endothelial cells [47] and FGF2-treated astrocytes [48]. Moreover, we observed that blockage of the ERK1/2 or JNK signaling pathway almost completely blocked EV-induced endothelial cell migration. Thus, our findings suggest that Egr-1 is a crucial proangiogenic transcription factor triggered by cancer-derived EVs and that ERK1/2 and JNK are upstream signaling pathways involved in EV-mediated endothelial Egr-1 activation. However, we could not completely exclude the possibility that other transcription factor(s) could be triggered by the ERK1/2 or JNK signal pathway. In the future, systemic analysis on EV-induced signaling pathways and transcriptional alternations should be conducted to identify the pivotal effectors in EV-induced angiogenesis comprehensively.

In summary, our study has revealed that Egr-1 activation in endothelial cells is a crucial mechanism of cancer-derived EV-induced angiogenesis. In addition, we showed that EV-mediated endothelial Egr-1 activation and migration were mediated by ERK1/2 or JNK signaling pathway and lipid raft-mediated endocytosis. Our findings improve our understanding regarding the angiogenic activities of cancer-derived EVs in the tumor microenvironment.
